# Endometriosis and the Temporomandibular Joint—Preliminary Observations

**DOI:** 10.3390/jcm12082862

**Published:** 2023-04-14

**Authors:** Małgorzata Wójcik, Tomasz Goździewicz, Zuzana Hudáková, Idzi Siatkowski

**Affiliations:** 1 Department of Physiotherapy, Poznan University of Physical Education, Faculty of Sport Sciences in Gorzow Wlkp., 61-871 Poznan, Poland; 2 Department of Perinatology and Gynecology, Division of Gynecology, Poznan University of Medical Sciences, 61-758 Poznan, Poland; 3 Faculty of Health, Catholic University, 034 01 Ružomberok, Slovakia; 4 College of Polytechnics, 586 01 Jihlava, Czech Republic; 5 SNP Central Military Hospital, Faculty Hospital, 034 01 Ružomberok, Slovakia; 6 Department of Mathematical and Statistical Methods, Poznan University of Life Sciences, 60-637 Poznan, Poland

**Keywords:** endometriosis, temporomandibular joint, interdisciplinary

## Abstract

(1) Background: The complete picture of the disease is not fully recognized and extends far beyond the pelvis. The disease’s impacts lead to systemic inflammation, in turn resulting in sensitization to pain. The aim of this study was to check whether statistical correlations exist in women with endometriosis with regard to their experience of pain: headache, pelvic pain, temporomandibular joint pain, along with teeth clenching and the treatment of the disease. We constructed contingency tables, followed by Pearson’s chi-square test and Cramer’s V coefficient values. (2) Methods: A survey was conducted among 128 women aged 33.43 ± 5.79 with a diagnosis of endometriosis (disease duration 6.40 ± 5.88 years). (3) Results: There was a correlation between the occurrence of pain on the right and left sides of the pelvis and pain on the right and left sides of the temporomandibular joint, *p*-value = 0.0397, V = 0.2350, and between the presence of pelvic pain and the treatment of endometriosis, *p*-value = 0.0104, V = 0.3709, and between the presence of pain outside the pelvis and the treatment of endometriosis, *p*-value = 0.0311, V = 0.4549. There was a highly significant correlation between teeth clenching and temporomandibular joint pain, *p*-value = 0.0005, V = 0.3695. (4) Conclusions: The study revealed a correlation between pelvic endometriosis symptoms and symptoms in the temporomandibular joint.

## 1. Introduction

Endometriosis is an estrogen-dependent inflammatory disease in which endometrial tissue appears outside its natural site [1]. The disease may have foci in the peritoneal cavity, ovaries, abdominal organs, or bladder. Foci may also occur in scars from episiotomy or caesarean sections and grow into the muscular wall of the uterus. The pathogenesis of endometriosis is multifactorial, and several theories have been put forward to offer an explanation. These include retrograde menstruation, coelomic metaplasia, embryological rest, and lymphovascular invasion. Hormones, immune status, and genetic factors may also play a role [2]. Clinical manifestations include pelvic pain due to dysmenorrhea or non-menstrual pelvic pain and infertility. Women often report difficulty articulating their symptoms or feel that their symptoms are inappropriately unified [3]. Women experience pelvic pain associated with endometriosis, for example, during intercourse, defecation, or micturition. During menstruation, the pain may intensify, which is related to hormonal changes [4]. In addition to severe pain [5], endometriosis is also a risk factor for malignancy, including endometrial cancer or clear cell carcinoma, which appear more frequently within endometriotic lesions of the ovary [2,6].

More than 176 million women worldwide have endometriosis [7]. Despite the very high prevalence of the disease, its diagnosis is unsatisfactory, and the time until it is diagnosed ranges from 4 to 11 years, with 65% of women initially misdiagnosed [3,8].

The risk of endometriosis is increased by family history [9], preterm birth [10], low birth weight [11], feeding infants with baby formula [10], reduced growth during childhood [12], painful first menstruation [13], low body weight—low BMI, freckles [14], miscarriages and abnormal pregnancy [15,16], and autoimmunological diseases [17].

Endometriosis is a disease in which pain occurs not only in but outside the pelvis. The presence of endometrium in the left temporal bone was observed to be associated with the presence of severe pain in the left temporomandibular joint [18]. In turn, the occurrence of pain in the temporomandibular joint is related to pain occurring in other parts of the body, e.g., hip, knee, ankle, shoulder, wrist, and elbow pain [19].

Temporomandibular joint (TMJ) dysfunctions often coexist with abnormal body alignment such as head protraction, deepened lumbar lordosis, or foot architecture abnormalities [20]. Patients with any TMJ dysfunction may also present with otological symptoms such as tinnitus, a sensation of fullness in the ear, ear pain, hearing loss, hypersensitivity, and dizziness. These complaints may be due to the anatomical proximity between the TMJ and the muscles and structures of the ear that are innervated by the trigeminal nerve [21]. Temporomandibular joint dysfunction is most commonly considered to be caused by malocclusion, neurological disorders, postural abnormalities, and traumatic changes [22].

Temporomandibular joint degenerative disease (TMJ-DD) is a condition that often occurs in women over 40 years of age and is associated with estrogen loss [23]. It has been proven that the fibrocartilage, which covers the articular surfaces, has estrogen receptors responsible for the normal trophism [24,25]. Women with endometriosis are often treated with hormonal therapy to put them into hypoestrogenism, the aim being to suppress their symptoms [26,27]. Progestins and low-dose oral contraceptives are ineffective in one-third of symptomatic women worldwide, which is probably the result of progesterone resistance [3].

It has also been proven that headaches, including migraine pain, are associated with painful temporomandibular disorders (TMD) [28]. It is often the case that women with endometriosis experience severe headaches, including even migraine-like headaches [29].

The current state of knowledge does not allow us to conclude unequivocally that, in women with endometriosis who experience pelvic and extra-pelvic pain, this pain is associated with the occurrence of pain in the temporomandibular joint. Nevertheless, endometriosis should be considered a systemic disease and not merely a gynecological disease affecting mainly the pelvis [3]. Endometriosis affects the metabolism in the liver and adipose tissue, leads to systemic inflammation, and alters the gene expression in the brain, resulting in sensitization to pain and mood disorders [3]. In gynecological diagnostics performed by specialists from various fields, a serious diagnostic problem can be the symptom of pain experienced by patients, which may be mixed pain. The definition of this pain is “a complex overlap of different known types of pain (nociceptive and neuropathic) in any combination, acting simultaneously and/or concurrently, causing pain in the same area of the body. Each of these mechanisms can be more clinically dominant at any given time. Mixed pain can be acute or chronic” [30]. Modern pain taxonomy has proposed the concept of nociplastic pain, which is defined as altered nociception, but the cause of pain is not tissue or somatosensory damage [31].

The full picture of the disease is not fully recognized and extends far beyond the pelvis. Treatment of endometriosis should be more comprehensive/interdisciplinary than that which is currently available. Therefore, the authors decided to check for any statistical correlations among women with endometriosis regarding the experience of pain: headache, pelvic pain, and temporomandibular joint pain, along with teeth clenching or the treatment of the disease.

## 2. Study Aim

The aim of this study was to determine whether statistical relationships exist related to musculoskeletal pain due to endometriosis. When formulating a question regarding pain sensations in the pelvis, in body parts outside the pelvis, e.g., abdomen, ribs, and in the TMJ, the question of whether the pain was of muscular or joint origin was not distinguished; only the occurrence of pain was analyzed.

The following research questions were posed:

1. Is there a correlation between the presence of pain on the right and left sides of the pelvis and pain on the right and left sides of the mandible (temporomandibular joint)?
2. Is there a correlation between the presence of pain in the pelvis (right, left, middle, and back) and headaches on the right and left sides (temples), middle (forehead), and back (occipital)?
3. Is teeth clenching related to headaches?
4. Is teeth clenching related to pain in the temporomandibular joint?
5. Is there a correlation between the occurrence of pain in the temporomandibular joint (TMJ) and the treatment of endometriosis?
6. Is there a correlation between the occurrence of pain in the pelvis and the treatment of endometriosis?
7. Is there a correlation between the occurrence of pain outside the pelvis (in other parts of the body) and the treatment of endometriosis?

## 3. Materials and Methods

This was an anonymous questionnaire study, approved by the Bioethics Committee of the Poznan University of Medical Sciences, protocol number KB—819/22, and conducted among 128 women aged 33.43 ± 5.79 with diagnosed endometriosis (disease duration 6.40 ± 5.88 years). All patients had endometriosis diagnoses in hospitals in the past. In was conducted according to current ESHRE guideline recommendations on how to diagnose endometriosis [32,33,34,35]. All patients had laparoscopy followed by medical treatment and then by combined hormonal contraceptives or progestogens for at least 6 months. We excluded patients with 3rd- and 4th-degree endometriosis. All patients included in the study declared that they had finished medical treatment for their endometriosis at least 6 months prior to the questionnaire.

Women with a medical diagnosis of endometriosis made by a gynecological specialist were requested to complete paper questionnaires.

At the time of completing the questionnaire, the women had not undergone any treatment intervention. The recruitment period was 3 months, filling out the questionnaires was random.

The inclusion criterion was diagnosed endometriosis.

Exclusion criteria for participation in the study were medical conditions: rheumatoid arthritis, lupus erythematosus, fibromyalgia, and a history of trauma(s) including pelvic injury and TMJ.

A total of 158 questionnaires were collected, of which 128 were analyzed on the basis of the adopted exclusion criteria. The data collected concerned the occurrence of musculoskeletal pain.

After data collection, a statistical analysis was performed using the R statistical package [36]. In order to answer the research questions posed and to test for the presence of relationships between variables, contingency tables were constructed, followed by Pearson’s chi-square test and Cramer’s V coefficient values [37]. A significance level of 0.05 was adopted.

## 4. Results

Statistical analysis provided results that, in part, confirmed the existence of statistical relationships between musculoskeletal pain in endometriosis (Table 1).

**Figure 1:**
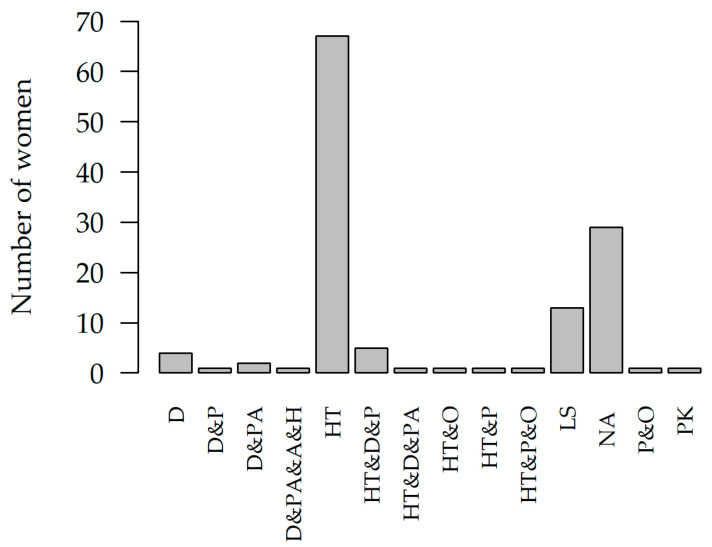
Treatments used among the subjects OX (D = Diet, PA = Physical Activity, P = Physiotherapy, O = Osteopathy, A = Acupuncture, PK = Pain Killer, HT = Hormonal Therapy, LS = Laparoscopy, H = Herbal, NA = Not Applicable, PS = Psychotherapy), OY (number of women undergoing treatment).

Statistical analysis yielded the following results in response to the questions posed, in the order listed above:

1. There was a correlation between the presence of pain on the right and left sides of the pelvis and pain on the right and left sides of the temporomandibular joint, *p*-value = 0.0397, V = 0.2350 (Table 1).
2. There was no relationship between the presence of pelvic pain on the right, left, middle, and back and headaches on the right and left sides (temples), middle (forehead), and back (occipital), *p*-value = 0.0647, V = 0.3469 (Table 1).
3. There was no relationship between teeth clenching and headache, *p*-value = 0.4715, V = 0.2886 (Table 1).
4. There was a highly significant relationship between teeth clenching and temporomandibular joint pain, *p*-value = 0.0005, V = 0.3695 (Table 1).
5. There was no relationship between the occurrence of temporomandibular joint pain and the treatment of endometriosis, *p*-value = 0.5214, V = 0.3274 (Table 1).
6. There was a correlation between the occurrence of pelvic pain and the treatment modality for endometriosis, *p*-value = 0.0104, V = 0.3709 (Table 1, Figure 1).
7. There was a relationship between the presence of pain outside the pelvis (in other parts of the body) and the treatment modality for endometriosis, *p*-value = 0.0311, V = 0.4549 (Table 1, Figure 1). All subjects reported that they clench their teeth; we did not assess anxiety/stress levels in this study.

Figure 1 Type of treatment used: diet, physical activity, physiotherapy, osteopathy, acupuncture, pain medication, hormone therapy, laparoscopy, herbal medicine, and psychotherapy. The predominant treatment modalities were hormone therapy and laparoscopy.

## 5. Discussion

The World Endometriosis Society reviewed studies on pain symptoms and the classification of endometriosis presented by the American Society for Reproductive Medicine (ASRM) and ENZIAN. The WES established that there is a great need to increase knowledge of this disease, as there is a lack of correlation between symptoms and pain [38,39,40,41]. ESHRE notes the development of quality indicators for the diagnosis and treatment of endometriosis [33].

Musculoskeletal disorders may also have symptoms that overlap with endometriosis. Therefore, the early recognition and treatment of endometriosis is essential [42]. Endometriosis does not have pathognomonic symptoms characteristic of a disease isolated in the pelvis. Rather, it has symptoms that are common to other gynecological and non-gynecological disorders [3]. Pelvic pain is the most distinct and problematic symptom coinciding with the observed location of the lesions [3]. The results obtained indicated that endometriosis is not a disease limited to the presence of pain only in the pelvis but that such pain also appears in the temporomandibular joints (*p*-value = 0.0397, V = 0.2350, Table 1). ESHRE recommendations indicate that clinicians should consider the presence of shoulder pain [32] and chest pain [34] in the diagnosis of endometriosis.

According to the US National Institutes of Health, the concept of chronic overlapping pain conditions (COPC) includes chronic fatigue syndrome, chronic (non-specific) sacral pain, chronic tension headache, endometriosis, fibromyalgia, migraine, painful bladder syndrome, vulvodynia, and temporomandibular disorders [43]. The symptoms observed in women with endometriosis indicate that the disease has a broad systemic impact, even though it is focused on pelvic lesions, primarily because of the frequent pain that occurs in this area and the lesions it causes. Endometriosis is not a disease limited to pelvic lesions [3].

However, the level of knowledge about endometriosis among women is insufficient, which may have an impact on the failure to see a specialist and thus delay the diagnosis of the disease and the initiation of treatment [44]. Although endometriosis may affect as many as 176 million women around the world, we still know little about the disease, and its causes are still being sought [45]. Most women with endometriosis report symptoms as early as puberty, and diagnosis is often delayed. Several years pass between diagnosis and treatment [46]. It would be important to implement endometriosis awareness campaigns among the public and develop interventions to treat and improve the quality of life of women with endometriosis [47]. ESHRE recommendations highlight strong and weak recommendations in the diagnosis of endometriosis [34].

Specific microRNA is a potential biomarker of endometriosis [48]. Recent studies have shown that microRNAs and their targeted microRNAs have different expressions in women suffering from endometriosis from those in healthy women [48]. MicroRNA controls a wide spectrum of normal and pathological cellular functions and, according to researchers, may play a key role in the pathogenesis of this disease [48]. However, the usefulness of microRNA biomarkers in detecting endometriosis is still uncertain [49], and the search for a biomarker or set of biomarkers remains open.

So far, no optimal way to diagnose endometriosis has been found, nor is there a single effective method for a full recovery. The main symptom influencing the quality of life of women with endometriosis is their perception of pain [50], and pharmacological treatment is the dominant treatment in endometriosis, which also aims to reduce pain [51]. In justified situations, surgery is the treatment of choice [52].

Women with endometriosis often experience severe headaches, even migraine headaches [28]. Such headaches are common in women with chronic pelvic pain, regardless of endometriosis [52]. However, Wu et al. demonstrated that, in women with endometriosis, migraine pain has a strong association with the disease [53]. In our study, there was no statistical relationship between the occurrence of headache and pelvic pain (*p*-value = 0.0647, V = 0.3469—Table 1) and headache and teeth clenching (*p*-value = 0.4715, V = 0.2886—Table 1).

Headaches, including migraines, often occur in women of childbearing age and change together with hormonal changes and life phases [54]. The literature indicates that headaches are common in women with endometriosis [29]. Chronic pelvic pain is a form of centralized pain in which the human body develops a low pain threshold [55]. In women with endometriosis, the acute pain associated with the disease may centralize within three to six months as the pain becomes chronic [56]. This condition is often accompanied by irritable bowel syndrome [55]. The treatment of pelvic pain requires cooperation between multiple specialists [55]. In the case of pelvic pain, in addition to the female reproductive organs, the pelvic floor muscles should be examined for tenderness or hypertonicity [55]. Palpation of the lumbar spine, sacroiliac joints, and pelvis should also be performed [56]. It is important to carry out the Carnett test to determine whether patients with pelvic pain have abdominal wall pain [56]. In patients with chronic pelvic pain, asymmetry in iliac crest height and lumbar conjunctival levels has been observed [57], which is significant with muscle-fascia bands when transferring tension in the body to locations distant from the dysfunction, including such pain sensations and in the TMJ, for example [58].

In contrast, a highly significant relationship was observed between teeth clenching and temporomandibular joint pain (*p*-value = 0.0005, V = 0.3695—Table 1). Stress is proven to be associated with TMJ pain [59]. However, stress/anxiety levels were not assessed in our study. TMJ pain is often present in people with bruxism [60]. In our study, the women did not declare that they had bruxism or TMJ trauma.

The connections between teeth clenching and pain in TMJ have been described at length in the literature [61,62,63]. However, women with chronic pelvic pain have a higher prevalence of depression, anxiety, and disturbed sleep [55]. It has been proven that TMJ dysfunctions in the context of musculoskeletal chains can influence postural dysfunction [22]. However, studies to date have shown that pelvic dysfunction, including sacroiliac joint dysfunction, affects the TMJ, and vice versa [22,64]. Endometriosis is associated with increased levels of reactive forms of oxygen and oxidation products, reduced antioxidant and detoxification enzymes, and deregulated iron metabolism. High levels of oxidative stress contribute to inflammation, extracellular matrix degradation, angiogenesis, and cell proliferation. Pain sensations associated with endometriosis are attributed to neurogenic inflammation and a feedback mechanism involving macrophages, pro-inflammatory cytokines, and prostaglandins [65]. It has been suggested that vitamins C and E should be supplemented in women with endometriosis in order to reduce oxidative stress and pelvic pain sensations [66].

Pro-inflammatory cytokines and changes in circulating immune cell populations have been observed in women with endometriosis, creating an extensive inflammatory environment that extends beyond the pelvis [67,68,69]. In addition, there are countless microRNAs circulating that modulate gene expression throughout the body [70,71]. Data from animal models have shown that cells from the endometrium can migrate to other organs, including the lung, liver, spleen, and brain [72]. This might be the reason for the occurrence of pain not only in the pelvis but also outside the pelvis.

Viewing the body in terms of tensegrity, i.e., a relationship based on the principle that structures remain in a close relationship that impacts on each structure (an increase in tension in one element brings about an increase in tension of the others) [22,73,74]. The human body functions as a whole to counteract the force of gravity. A key role in this is played by the fascia wrapping around the internal organs in the same way as a scaffold and the transmission of tension [22]. Proinflammatory cytokines have been observed among individuals with myofascial pain [75]. The occurrence of pain in women with endometriosis stems from increased cytokine levels due to myofascial pain. Myofascial pelvic pain refers to pain in the pelvic floor muscles, the pelvic floor connective tissue, and the surrounding fascia [76]. Myofascial pain syndrome (MPS) occurs in women with endometriosis. This may be accompanied by the presence of trigger points in the pelvic floor muscles [77].

Patients with chronic pain have an increased risk of developing chronic pain syndrome with bladder dysfunction, irritable bowel syndrome, and vulvodynia [78]. Functional MRI assessments have revealed morphological adaptations in the brain after experiencing pain for a period of two years [46]. This often explains the severe pain that accompanies patients, even in the so-called absence of pathological changes. The pathogenesis of endometriosis-related pain is highly complex and is certainly not fully understood [46].

Common treatments for endometriosis include hormone therapy and surgery to treat the symptoms of pelvic disease. However, both approaches are associated with failure and do not provide a complete solution to the systemic effects of the condition [3]. Among the study participants, hormonal and laparoscopic treatments were used (Figure 1). Additional treatment among those surveyed were diet, physical activity, physiotherapy, osteopathy, acupuncture, pain medication, herbal medicine, and psychotherapy were also used in the treatment of the women studied (Figure 1). The statistical analysis showed that there was a statistical relationship between the type of treatment and the occurrence of pelvic (*p*-value = 0.0104, V = 0.3709 Table 1, Figure 1) and extra-pelvic (*p*-value = 0.0311, V = 0.4549 Table 1, Figure 1) pain. Bearing in mind the current state of knowledge of endometriosis, it is difficult to explain these findings.

Hormonal and laparoscopic treatment were the treatment modalities used among the respondents.

The former aims to put the body into a state of progesterone dominance [50,79].

Surgical treatment, depending on the surgical technique selected, leaves a scar in and on the patient’s body, which also requires physiotherapy intervention in the form of mobilization [80,81,82,83]. The surgical treatment used is challenging and still debatable, as pain relief after removal of endometrial tissue has not been demonstrated [84,85,86].

In addition to hormonal and laparoscopic treatment, the treatment methods used by the women were diet, physical activity, physiotherapy, osteopathy, acupuncture, painkillers, and herbal and psychotherapy.

The study confirms the effectiveness of using diet, physiotherapy, acupuncture, and herbal therapy in the treatment of endometriosis with reference to reducing pain in the body [4,87,88,89]. From the field of physiotherapy, Transcutaneous Electrical Nerve Stimulation (TENS) electrostimulation is the most frequently used pain relief treatment for endometriosis. TENS electrostimulation is a good method for reducing chronic pelvic pain (CPP) and deep dyspareunia, thereby improving women’s quality of life and sexual function [90]. An exercise-based ‘Phys-io-EndEA’ program may help to improve HRQoL in women with symptomatic endometriosis [91]. It is also important to bear in mind that this treatment is associated with high costs. In view of the high cost of treatment in each country, it is certainly the case that non-pharmacological methods/procedures to reduce pain among women with endometriosis and thus physiotherapeutic methods/procedures to improve the quality of life of women can be a good solution from the economic viewpoint of medical care but especially for the affected women.

The most common treatment for women with endometriosis is pharmacological treatment. Despite its widespread use for pelvic pain caused by endometriosis, there is a lack of evidence of the efficacy of NSAIDs [92]. During endometriosis, due to chronic pelvic pain, women often experience an unfavorable psychological state, depressive states, anxiety symptoms, and psychosomatic disorders [93]. Due to the complexity of the disease, psychotherapy is also suggested [94,95]. In the case of physical activity, no studies have been reported to show that physical activity reduces the pain sensations associated with endometriosis [96]. Moreover, in the field of osteopathy, few studies can uniquely demonstrate its effectiveness in endometriosis treatment [97].

We did not note any relationship between the occurrence of temporomandibular joint pain and the treatment of endometriosis, *p*-value = 0.5214, V = 0.3274 (Table 1). Based on the lack of scientific reports, it is difficult to comment on this result. However, when TMJ pain is present, a common treatment undertaken is pharmacotherapy, including botulinum toxin type A and physiotherapy [98,99,100]. Souza et al. demonstrated that patients with temporomandibular diseases (TMD) present differences in foot pressure in a pedobarographic examination compared to those without TMJ dysfunction [101]. Even with several treatments, it is not possible to cure the disease completely [102]. Endometrial lesions may persist in the body and recur after treatment ceases [102]. Therefore, it is important to seek new therapeutic alternatives in the treatment of endometriosis [102].

A study by Volker et al. showed that women with endometriosis who experienced pelvic pain rated themselves as less attractive, indicating a correlation between pelvic pain and negative body image presented by these patients [103].

The treatment of endometriosis will need to address the disease as a whole, including all chronic and systemic symptoms [3].

The results obtained here indicate a link between TMJ pain and the pelvis minor. Thus, it would be advisable for further research to be conducted by various specialists in order to search for ways to alleviate pain in the female body in the case of a disease such as endometriosis. Further extended research by the present authors may provide more answers about the incidence of musculoskeletal pain versus endometriosis. Due to the complexity of the disease, collaboration between medical professionals from different specialties would be advisable.

It is also important to remember that dispersed endometrial tissues cause disruptions in hormonal and immune processes in the organism, which may, in turn, increase susceptibility to the SARS-CoV-2 infection [100]. The COVID-19 pandemic led to an increase in symptoms such as painful menstruation, pelvic pain, anxiety, depression, and fatigue in women with endometriosis [104]. In our study, we did not collect data on the incidence and course of COVID-19 among the women interviewed. In addition to systemic diseases caused by SARS-CoV-2 virus, the occurrence of musculoskeletal pain after COVID-19 (‘Long COVID’) has also been observed [105]. The mechanism of musculoskeletal pain in ‘Long COVID’ is yet to be investigated [105]. Furthermore, the COVID-19 pandemic revealed an adverse effect on the psycho–emotional state of the populace, with exacerbated feelings of stress, anxiety, or depression. In turn, these can lead to increased temporomandibular diseases (TMD) and bruxism symptoms and increased orofacial pain [106]. It should be noted that during the COVID-19 pandemic, patients were subjected to virtual or remote health consultations.

## 6. Conclusions

This study reveals correlations between the presence of pain on the right and left sides of the pelvis and pain on the right and left sides of the temporomandibular joint, teeth clenching and temporomandibular joint pain, the occurrence of pelvic pain and the treatment modality for endometriosis, and the presence of pain outside the pelvis (in other parts of the body) and the treatment modality for endometriosis.

The study did not reveal a correlation between the presence of pelvic pain on the right, left, middle, and back and headaches on the right and left sides (temples), middle (forehead), and back (occipital); teeth clenching and headache; the occurrence of temporomandibular joint pain and the treatment of endometriosis.

### Limitations of the Study

A limitation of our study is that it was a one-time study and survey. Other limitations are the small number of women participating; and the fact that we did not address the BMI, gravida, and para values of the patients; the lack of information of the names of the hormonal drugs taken by the patients and exactly how long they took them (we only summarized that the women were treated with combined hormonal contraceptives or progestogens for at least 6 months).

However, this survey did indicate the need to implement multidisciplinary treatment in women with endometriosis in order to reduce the pain present in the body, not only in the pelvis but also in the temporomandibular joint.

## Figures and Tables

**Table 1:** Values from the statistical analysis (values: *p*-value < 0.05 and Cramer V).

Number of Research Question	*p* = Value	Cramer V
1.	**0.0397**	0.2350
2.	0.0647	0.3469
3.	0.4715	0.2886
4.	**0.0005**	0.3695
5.	0.5214	0.3274
6.	**0.0104**	0.3709
7.	**0.0311**	0.4549

## Data Availability

Data are available upon request.
